# Identification and Validation of an EMT-Related LncRNA Signature for HNSCC to Predict Survival and Immune Landscapes

**DOI:** 10.3389/fcell.2021.798898

**Published:** 2022-01-20

**Authors:** Chunyu Feng, Shaopeng Liu, Zhengjun Shang

**Affiliations:** ^1^ The State Key Laboratory Breeding Base of Basic Science of Stomatology (Hubei-MOST) and Key Laboratory of Oral Biomedicine Ministry of Education, School and Hospital of Stomatology, Wuhan University, Wuhan, China; ^2^ Department of Oral and Maxillofacial Head and Neck Oncology, School and Hospital of Stomatology, Wuhan University, Wuhan, China

**Keywords:** HNSCC, EMT, lncRNAs, prognostic signature, immunotherapy, TCGA

## Abstract

Long noncoding RNAs (lncRNAs) are increasingly recognized as decisive factors in the progression of head and neck squamous cell carcinoma (HNSCC), and they participate in the epithelial–mesenchymal transformation (EMT) of HNSCC. LncRNAs are closely related to the prognosis of patients with HNSCC; thus, it is essential to identify EMT-related lncRNAs with prognostic value for HNSCC. The coexpression network of EMT-related lncRNAs was constructed using The Cancer Genome Atlas (TCGA). An EMT-related eight-lncRNA-based prognostic signature was constructed using LASSO Cox regression and Cox proportional hazards analyses. Univariate and multivariate analyses and stratified prognosis confirmed that the prognostic signature was an independent predictive factor. Subsequently, we performed immune cell infiltration analysis, gene set enrichment analysis (GSEA), and single-sample GSEA (ssGSEA) pathway enrichment analysis to uncover the potential molecular mechanisms of prognostic differences in the high- and low-risk groups. Next, we discussed the relationship between the prognostic signature and immune checkpoint-related genes, their TIDE scores, and the sensitivity of common chemotherapeutics. Finally, we further verified the expression differences in lncRNAs that were included in our signature via RT–qPCR in eighteen paired tissues. In summary, this prognostic signature provides powerful prognostic biomarkers for HNSCC and could serve as a predictor for the sensitivity of common chemotherapeutics and immunotherapy responses as well as providing a reference for further personalized treatment.

## Introduction

Human head and neck squamous cell carcinoma (HNSCC) is one of the most common malignant tumors and the most crucial pathological type of head and neck cancer, with an incidence of approximately 600,000 new cases yearly (Bray et al., 2018). The prognosis of HNSCC is poor due to high proliferation, regional lymph node metastasis, and a high recurrence rate; the 5 years survival rate of HNSCC is nearly 50% (Burusapat et al., 2015; Lambert et al., 2017). The prognosis of HNSCC patients is still unsatisfactory, although treatment methods have been improved in recent decades (Yi et al., 2020).

The first step in metastasis of cancer cells is achieving local invasiveness. To that end, cancer cells must abandon epithelial phenotypes and acquire mesenchymal morphological and transcriptional characteristics. This process is usually called the epithelial–mesenchymal transformation (EMT). EMT is crucial for physiological and pathological processes, such as embryonic development, wound healing, and metastasis of malignant tumors (Lamouille et al., 2014; Nieto et al., 2016). During EMT, there is separation between epithelial cells and between epithelial cells and the basement membrane (Nieto et al., 2016). EMT is essential for metastasis and the acquisition of drug resistance. Nearly 90% of cancer patients die from metastasis rather than primary tumors. Therefore, EMT has an important effect on the prognosis of HNSCC patients. Nevertheless, at present, the mechanism of EMT has not been fully elucidated.

Long noncoding RNAs (lncRNAs) are defined as transcripts of more than 200 nucleotides that are not translated into proteins (Xing et al., 2019; Guo et al., 2020). LncRNAs are crucial for cellular processes. An increasing number of studies have shown that lncRNAs have a regulatory effect on EMT (Lu et al., 2017; Jia et al., 2019). The function of lncRNAs in regulating cell fate has received widespread attention. The role of lncRNAs has been reported in multiple cancers, such as lung cancer, gastric cancer, bladder cancer, and colorectal cancer (Lv et al., 2017; Zhang et al., 2018; Wang et al., 2019a; Bure et al., 2019). Nevertheless, the mechanism of lncRNAs and EMT and the role of lncRNAs in the prognosis of HNSCC have still not been elucidated.

EMT is indispensable for immunosuppression, immune tolerance, evasion, and metastasis (Dongre et al., 2017; Mittal, 2018). EMT influences the prognosis of patients, simultaneously providing an approach to discovering novel therapeutic targets and predictive biomarkers for tumor treatment (Jiang and Zhan, 2020). Immune cells occupy a large proportion of the tumor microenvironment of HNSCC. Adaptive immune resistance induces an immunosuppressive tumor environment that enables immune evasion (Srinivasan et al., 2018). This capability likewise enhances tumor development and metastasis. Immunotherapy, a neoteric treatment strategy for tumors, has been widely studied in recent years, and the results pertaining to immune checkpoint inhibitors (ICIs) are particularly satisfactory. Tumor cell-derived PD-L1 contributes to EMT and tumor invasion in multiple tumor types (Dong et al., 2018). Anti-PD-1 and anti-CTLA4 strategies occupy the forefront of the immunotherapy field. Tumor stroma composition and immune cell infiltration have a profound impact on the prognosis of patients (Galon et al., 2006; Dongre et al., 2017). Reduced stromal composition indicates an increase in cancer cells, and less immune cell infiltration indicates a reduced response to immunotherapy, which would predict a worse prognosis.

The treatment of HNSCC is mainly surgery combined with radiotherapy and chemotherapy. Clinical doctors generally predict patient prognosis based on the TNM stage or clinical stage; however, this method is not sufficiently accurate. A novel prediction method urgently needs to be established for the clinic. Therefore, in this study, we established a new method to diagnose early disease and predict prognosis accurately. To this end, we further explored the regulatory mechanism of EMT in HNSCC and found potential effective biomarkers for early diagnosis and accurate prognosis. HNSCC RNA sequencing (RNA-seq) data and clinical data were downloaded from The Cancer Genome Atlas (TCGA) database, and EMT-related genes were obtained from the study of Choi et al. (Choi et al., 2010; Yang et al., 2018). Univariate Cox regression analyses were used to identify key EMT-related lncRNAs. These lncRNAs were used to construct a prognostic signature with LASSO regression analysis (Vrieze, 2012). We divided the patients into high-risk and low-risk groups on the basis of our signature (Jiang et al., 2020). To evaluate the prognostic accuracy of this signature, we plotted Kaplan–Meier survival curves and receiver operating characteristic (ROC) curves and performed principal component analysis (PCA). We built a coexpression network to predict the function and mechanism of these lncRNAs. Univariate and multivariate Cox regressions were employed to appraise the independence of the signature. MultiROC curves were used to assess accuracy among multiple factors. A nomogram was used to predict the survival rate via multiple independent factors. Then, we compared differences between the two groups in terms of immune infiltration, sensitivity to frequently used chemotherapeutic drugs, and gene set enrichment. Finally, we verified lncRNA expression differences among eight paired paracarcinoma tissues and cancer tissues.

## Materials and Methods

### Data Collection and Preparation

RNA-seq data and clinical data were downloaded from the TCGA database (https://portal.gdc.cancer.gov/) (Yang et al., 2018). Fragments per kilobase million (FPKM) values were employed in the next analysis. The samples in this study, mostly from the tongue, oral floor, and cheek, included 44 normal structures and 525 malignant tumors from TCGA. The RNA-seq data and clinical data were combined by using R (R version 4.0.4). Protein-coding genes and lncRNAs were annotated and classified using the Ensemble human genome browser GRCh38.p13 (http://asia.ensembl.org/index.html) (Sun et al., 2020). This study excluded repeated samples and samples for which clinical data were not known.

### Identification of Prognostic EMT-Related Hub LncRNAs

We used TCGA data to identify lncRNAs associated with EMT-related genes via the R software package “limma.” EMT genes were obtained from the study of Choi et al. (Choi et al., 2010). Pearson’s correlational coefficients assessed the association of lncRNAs and EMT-related genes. The threshold value of Pearson’s correlational coefficient was >0.3 or < −0.3, and the *p* value was <0.01, in the identification of the differentially expressed EMT-related lncRNAs of tumor tissue and normal tissue. The association of EMT-related lncRNA expression and prognosis was assessed by univariate Cox regression, and the overall survival rate was calculated using the R software package “survival.” A *p* value <0.001 indicated a significant difference.

### Estimation of the Prognosis Signature

The data from TCGA were divided randomly and equally into two groups: a training set (*n* = 251) and a validation set (*n* = 248). We adopted LASSO regression via the R software package “glmnet” to filter prognosis-related lncRNAs. ROC curves were applied to verify this signature (Zhang et al., 2018). Then, the validation set further validated the signature. Risk scores of every patient from total TCGA were calculated (Yoshihara et al., 2013). Then, all HNSCC patients were divided into a high-risk group (*n* = 254) and a low-risk group (*n* = 245) based on the median risk score. Kaplan–Meier survival curves were used for eight EMT-related lncRNAs to explore the relationship between prognosis and lncRNAs. PCA was used to evaluate the expression profile between the two groups.

### Validation of the Independence and Forecast Efficiency of the Prognostic Signature

Univariate and multivariate Cox analyses were utilized to discover independent risk factors among age, sex, grade stage, clinical stage, TNM stage, and risk score. MultiROC curves were employed to compare efficiency among selected risk factors.

### CIBERSORT Analysis of Immune Condition and Function

We employed the CIBERSORT database to predict the differences among 22 immune cells in infiltration conditions of two groups (https://cibersort.stanford.edu/) (Newman et al., 2015). The algorithm of 100 permutations was adopted. Only samples with a *p* value <0.05 were included to perform the subsequent analysis comparing differential immune infiltration levels between two groups grouped by clustering subtypes and risk scores.

### ssGSEA Assessment of Tumor-Infiltrating Cells

Bindea et al. found numerous marker genes of tumor-infiltrating immune cells (TIICs) (Angelova et al., 2018). Using ssGSEA, the enrichment scores of 16 immune cells and 13 immune functions for each HNSCC sample were quantified based on gene signatures using the R software package “gene set variation analysis” (GSVA) (Hänzelmann et al., 2013). The immune infiltration levels and functions between the two groups were compared by the Kruskal–Wallis test. ESTIMATE was used to evaluate the tumor microenvironment and predict the tumor purity and abundances of tumoral stromal cells based on the gene expression data of the high-risk group and low-risk group (Jiang et al., 2020).

### Evaluation of Immunotherapy and Drug Sensitivity Prediction

We further assessed the response to immune checkpoint inhibitors (ICIs), such as CTLA4 and PD-1. The response to ICI was predicted by the Tumor Immune Dysfunction and Exclusion (TIDE; http://tide.dfci.harvard.edu/). The differences between the two groups were discovered by the Wilcoxon test. Furthermore, the therapeutic effects of anti-PD1 and anti-CTLA4 were predicted for HNSCC patients in each of the two groups. The sensitivity of each patient to commonly used chemotherapy drugs for HNSCC was estimated using the Genomics of Drug Sensitivity in Cancer (GDSC; https://www.cancerrxgene.org/) database (Yang et al., 2013). The half-maximal inhibitory concentration (IC50) was quantified through the R software package “pRRophetic” (Geeleher et al., 2014).

### Gene Set Enrichment Analysis

In terms of the signaling pathway analysis, differential expression analysis was first conducted on all genes of the samples with high- and low-risk scores by applying the R software package “limma.” Gene Ontology (GO) and Kyoto Encyclopedia of Genes and Genomes (KEGG) analyses with GSEA software were performed on the differentially expressed genes (DEGs) between the high-risk and low-risk groups to research the signaling pathways in which DEGs are involved. (*p* < 0.05 and false discovery rate, FDR <0.25).

### Deciphering Mutational Landscape in the Genome

Mutation data of patients with HNSCC were downloaded from the TCGA database. The top 20 driver genes with the highest frequency of change were further analyzed. Oncoplots were sketched between high- and low-risk groups by R software “maftools.”

### Verification of Risk Signature Stability and Prediction of Survival Rate

Kaplan–Meier survival curves were employed to analyze the stability of the signature among imparity clinical features, including age and sex. We ultimately structured a nomogram to predict the survival rate accounting for the independent features.

### Verification of Expression Differences

LncRNAs incorporated into signature expression differences of eighteen paired paracarcinoma tissues and cancer tissues were verified by qRT–PCR. The primers used for qRT–PCR are summarized in Table 1. Clinical data of all patients recruited were showed in Supplementary Table S1. According to the manufacturer's instructions, total RNA was extracted and reverse transcribed from tissue from eighteen patients by a Takara Reagent kit. The SYBR Master Mixture Kit (Takara) was utilized to amplify the resulting cDNA, and qRT–PCR was performed using a QuantStudio(TM) 6 Flex System according to the manufacturer's instructions. Each experiment was conducted at least three times. The expression of target lncRNAs was calculated using the 2-∆Ct method relative to the internal reference (ACTB).

**TABLE 1:** Primers of LncRNAs in prognosis signature.

AL138902.1	ENSG00000234779	Forward	TCA​GAG​ACC​AGC​CTT​CGA​AG
		Reverse	AGG​ACT​GAG​GCT​GTG​TTC​TC
AL356481.3	ENSG00000280758	FORWARD	GAG​CCA​ATA​TCG​CAC​CAC​TG
		REVERSE	CCC​TTC​CCT​TCC​TGG​CTT​AA
AC024267.3	ENSG00000264304	FORWARD	GTT​TCA​CCT​TGT​CAG​CCA​GG
		REVERSE	GCT​TCC​TGT​TGT​TGC​TAC​CC
LINC00996	ENSG00000242258	FORWARD	GAG​GGC​ACT​TTG​TCT​TAC​TTG​GC
		REVERSE	ATT​CTT​CAT​GCC​AAT​CCT​CTC​AC
AC245041.2	ENSG00000276850	FORWARD	AGT​GAA​AGA​GAT​GGC​CTG​GT
		REVERSE	TCC​TGG​GAG​TCG​TTA​GAA​GC
AL596223.1	ENSG00000229261	FORWARD	GCC​AAA​GTT​CTT​GTC​CCC​AG
		REVERSE	CAG​TGA​GTC​CCC​TTC​TTC​GT
AP003774.4	ENSG00000239650	FORWARD	CGC​CCC​AGA​GAT​AGT​GAC​AT
		REVERSE	AGC​TGT​CAG​GTA​TTG​TGG​CT
AC114730.3	ENSG00000235351	FORWARD	GGA​AAC​ATC​CAG​CGC​TTC​AA
		REVERSE	CGC​CTT​TCT​TGT​CAC​AGG​TT

### Statistical Analysis

All data were analyzed by R software. A *p* value <0.05 was considered a significant difference. The independent Student’s t test for continuous data and the χ^2^ test for categorical data were utilized for pairwise comparisons between groups. The Wilcoxon test was used to compare non-normally distributed variables between two groups. The log-rank test was used to compare two groups when Kaplan–Meier survival curves were generated.

## Results

### Identification of EMT-Related LncRNAs

**FIGURE 1:**
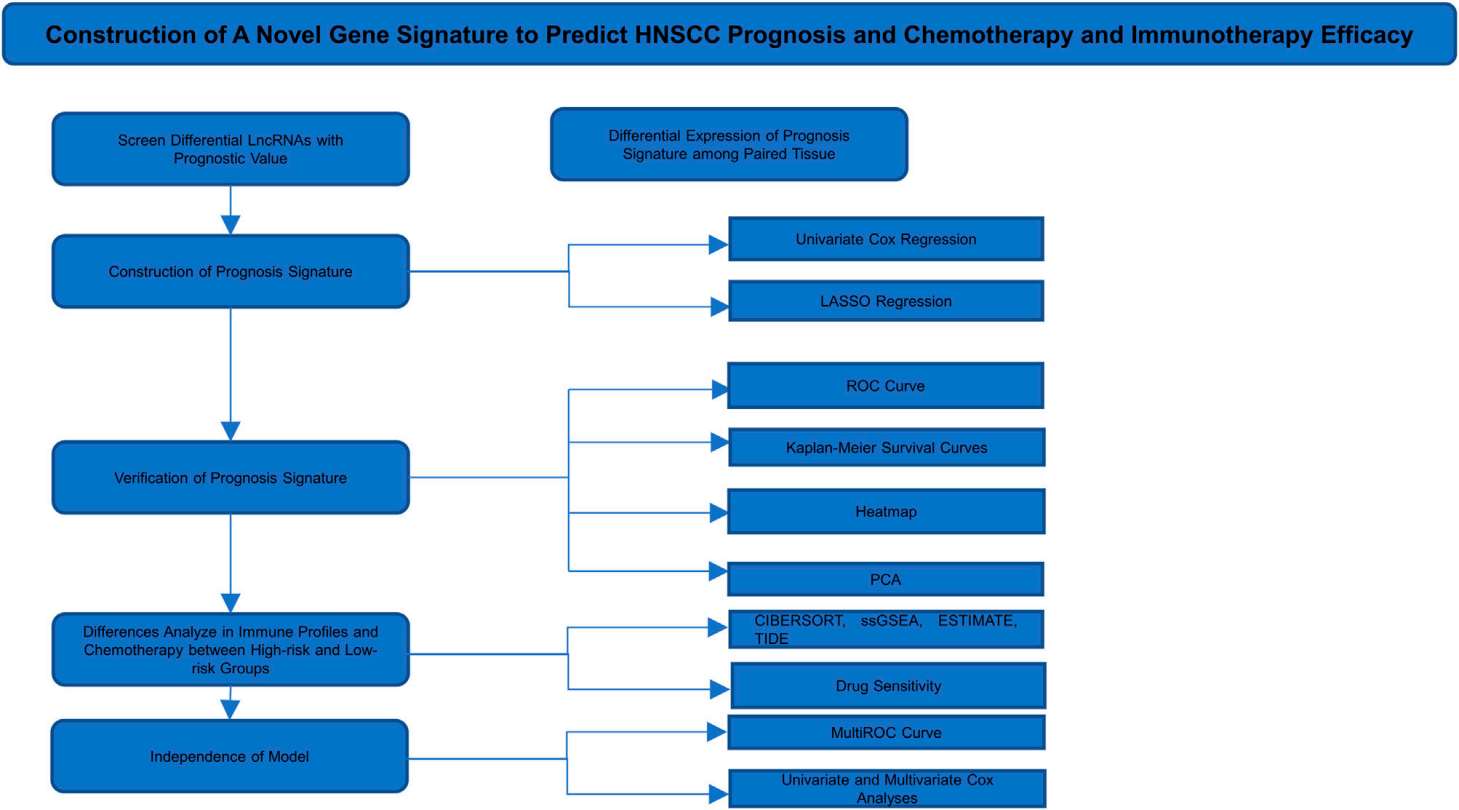
Flow chart of the study design.

A total of 14,142 lncRNAs were identified by analyzing RNA-seq data from HNSCC patient tissue samples in the TCGA database and 200 EMT-related genes. A total of 1,598 lncRNAs were screened by calculating Pearson’s correlation coefficients between the lncRNAs and EMT-related genes. The identified lncRNAs were defined as EMT-related lncRNAs. We conducted our study as shown in Figure 1.

### Establishment of an EMT-Related Eight-lncRNA Prognostic Signature

**FIGURE 2:**
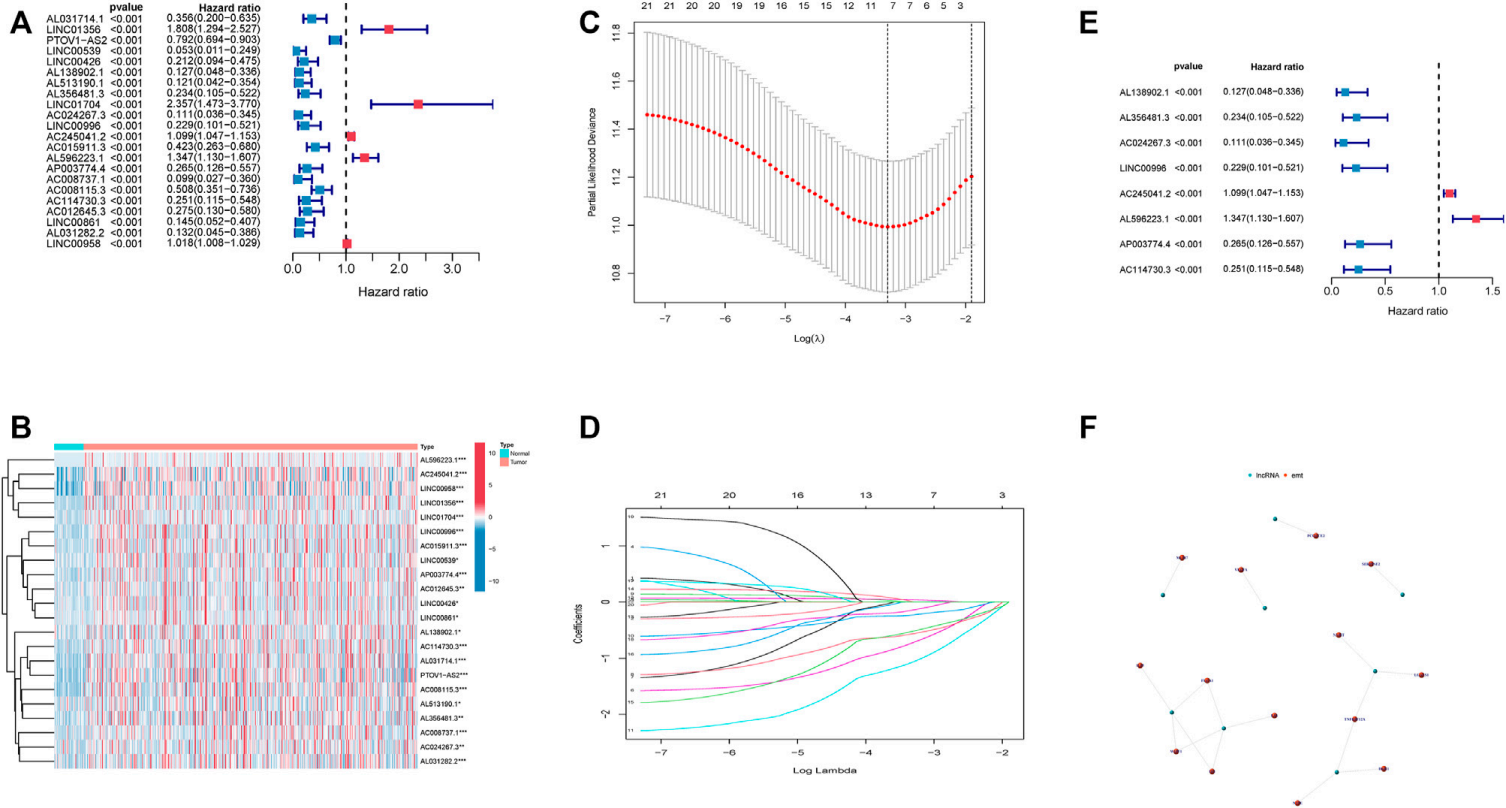
Identification of an 8 EMT-related lncRNA prognostic signature. **(A)**. Univariate Cox regression analysis of 22 EMT-related lncRNAs. **(B)**. Heatmap of 22 EMT-related lncRNAs. **(C)**. Partial likelihood deviance was plotted versus log(λ). **(D)**. LASSO coefficient profiles of lncRNAs associated with overall survival of HNSCC. **(E)**. Forest plot depicting associations between 8 lncRNAs that were screened by LASSO regression analysis and risk value. **(F)**. network of 8 lncRNA and related genes.

Out of 1,598 EMT-related lncRNAs, 22 lncRNAs were selected through univariate Cox regression analysis and were associated with the prognosis of HNSCC patients (Figures 2A,B), and the *p* value was <0.001. Patients were randomly and equally divided into training and validation sets. LASSO regression analysis was conducted to assess the correlations between these 22 lncRNAs and survival status in the training set (Figures 2C,D). Then, we included eight lncRNAs as a predictive prognostic signature. Of the 8 lncRNAs, 6 lncRNAs were protective factors (hazard ratio, HR < 1), and 2 lncRNAs were risk factors (HR > 1) (Figure 2E). The network of 8 EMT-related lncRNAs and EMT-related genes was revealed by a network diagram (Figure 2F). Only AL138902.1 negatively regulated the downstream genes LGALS1, NNMT, and TNFRSF12A in 18 pairs of interactions.

### Evaluation of the Prognostic Signature

**FIGURE 3:**
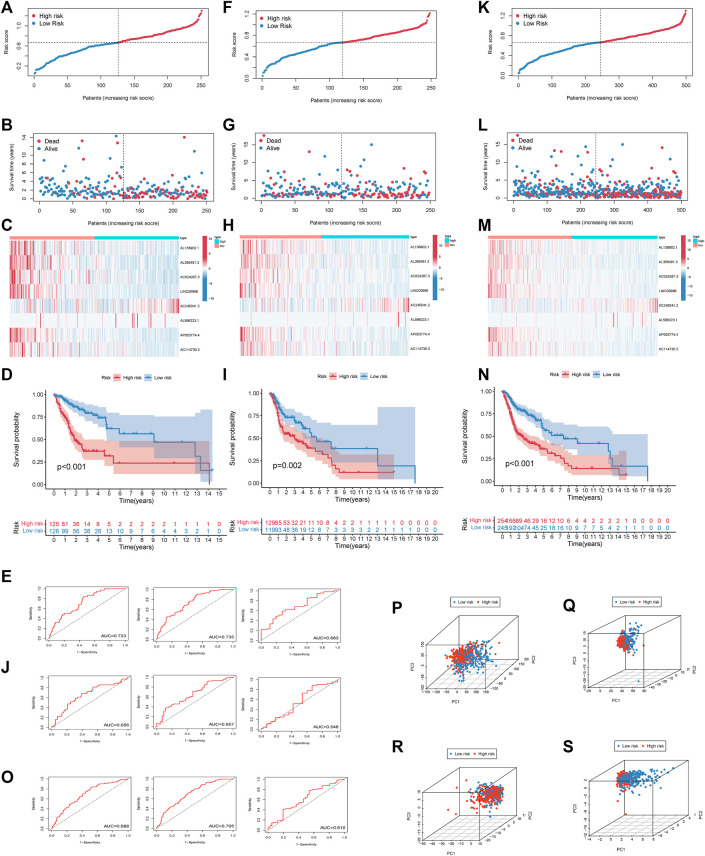
Estimation of the prognosis model of 8 EMT-related lncRNAs. **(A)**. The survival rate was significantly associated with the risk score in the training set. **(B)**. Scatterplot of patient survival in the training set. **(C)**. Heatmap of 8 EMT-related lncRNAs in the training set. **(D)**. Kaplan–Meier survival curves of the high-risk group and the low-risk group in the training set. **(E)**. ROC curve based on the risk score model in the training set. **(F)**. The survival rate was significantly associated with the risk score in the test set. **(G)**. Scatterplot of patient survival in the test set. **(H)**. Heatmap of 8 EMT-related lncRNAs in test set **(I)**. Kaplan–Meier survival curves of the high-risk group and the low-risk group in the test set. **(J)**. ROC curve based on the risk score model in the test set. **(K)**. The survival rate was significantly associated with the risk score in all sets. **(L)**. Scatterplot of patient survival in all sets. **(M)**. Heatmap of 8 EMT-related lncRNAs in all sets. **(N)**. Kaplan–Meier survival curves of the high-risk group and the low-risk group in all sets. **(O)**. ROC curve based on the risk score model in all sets. Principal Component Analysis Verifies the Performance of The Risk Model (all genes, EMT-related genes, EMT-related LncRNAs, 8 EMT-related LncRNAs). Red and blue dots represent high-risk and low-risk patients, respectively **(P–S)**.

Risk scores of each HNSCC patient were calculated, and the patients were divided into high-risk and low-risk groups based on the median risk score in the training set. A correlation diagram between the survival rate and risk score (Figure 3A), a scatterplot of the patients (Figure 3B), a heatmap (Figure 3C), Kaplan–Meier survival curves (Figure 3D), and 1, 3, and 5 years survival ROC curves (Figure 3E) were used to evaluate this signature. The validation set and all sets were used to further verify the accuracy of this signature (Figures 3F–O). HNSCC patients were sorted according to their risk scores, and the survival rate was significantly associated with the risk score. The survival time of patients in the high-risk group was lower than that of patients in the low-risk group. This result suggested that the higher the score was, the worse the prognosis. In the heatmap, the expression of 8 EMT-related lncRNAs was remarkably different between groups. The expression of AL138902.1, AL356481.3, AC024267.3, LINC00996, AC114730.3, and AP003774.4, as risk factors, was higher in the high-risk group than in the low-risk group. On the other hand, the protective factors AC245041.2 and AL596223.1 were expressed in the opposite direction. According to the Kaplan–Meier survival curves, patients with low-risk scores survived notably longer than those with high-risk scores. ROC curves were plotted to assess the predictive value of the prognostic signature. The training set AUC values of the 1, 3, and 5 years ROC curves were 0.723, 0.735, and 0.683, respectively, and the AUC values of the 1, 3, and 5 years ROC curves of all patients were 0.688, 0.705, and 0.610. These results confirmed that our signature had medium accuracy for evaluating the prognosis of HNSCC patients. PCA was conducted to investigate the distributional differences based on different patterns (Figures 3P–S). We found that eight EMT-related lncRNAs were better prognostic genes by which high- and low-risk patients could be more easily separated than risk genes, EMT genes, and all risk genes.

**FIGURE 4:**
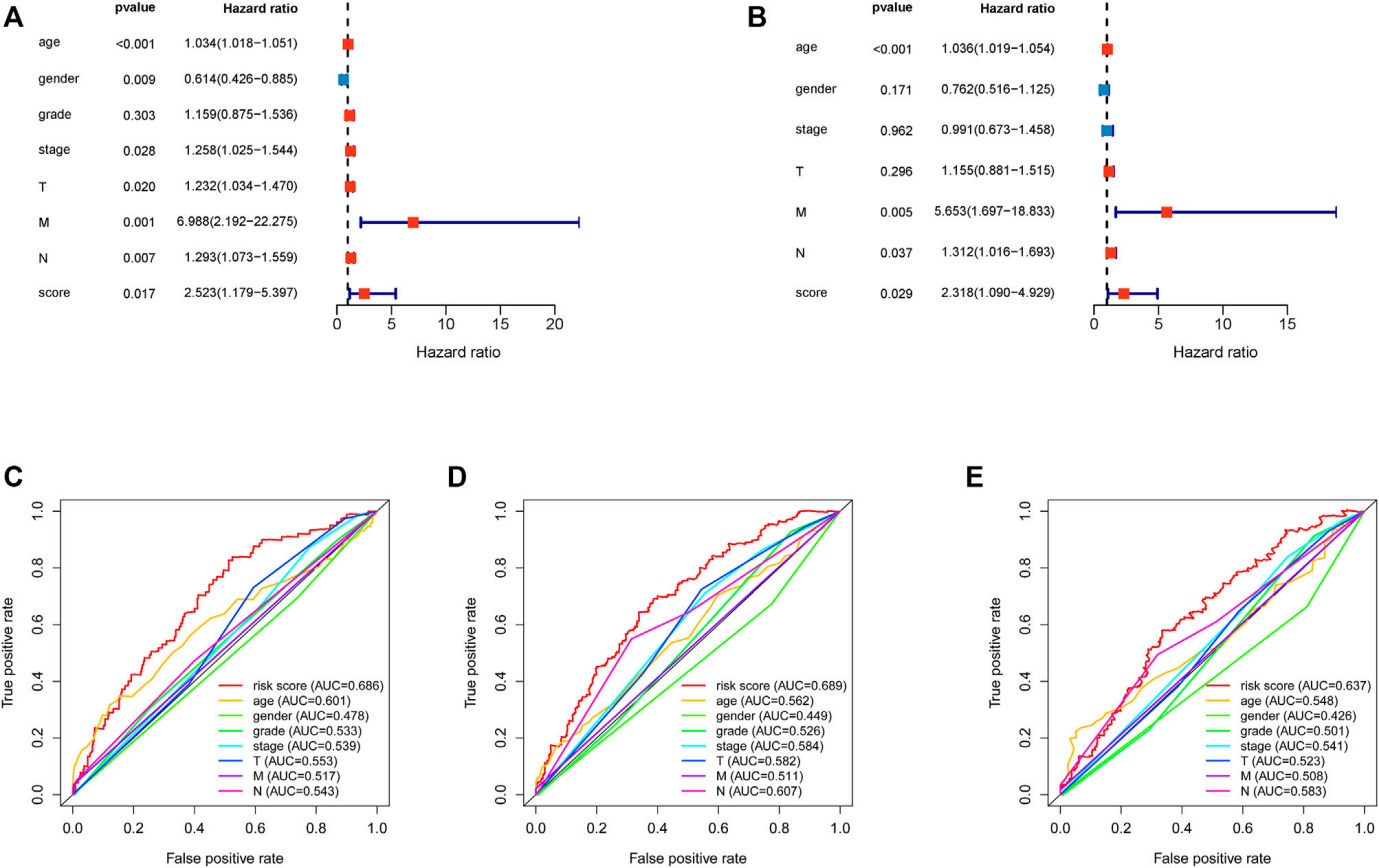
Assessment of the independence of the HNSCC prognostic model and comparison between risk score and clinical features. **(A)**. Univariate analysis Cox regression analysis confirmed the independence of the prognostic model. **(B)**. Multivariate Cox regression analysis confirmed the independence of the prognostic model. HNSCC, head and neck squamous cell carcinoma. **(C)**. 1 year multiROC curve of risk score and clinical features. **(D)**. 3 year multiROC curve of risk score and clinical features. **(E)**. 5 year multiROC curve of risk score and clinical features.

Univariate and multivariate Cox regression analyses demonstrated that the risk score was an independent predictor of prognosis in HNSCC patients, as were age, M stage, and N stage (Figures 4A,B). Furthermore, the efficacy of the risk score was higher than that of other clinical factors at 1 year (Figure 4C), 3 years (Figure 4D), and 5 years (Figure 4E) ROC curves.

### Tumor Immune Microenvironment Characteristics of Different Groups

**FIGURE 5:**
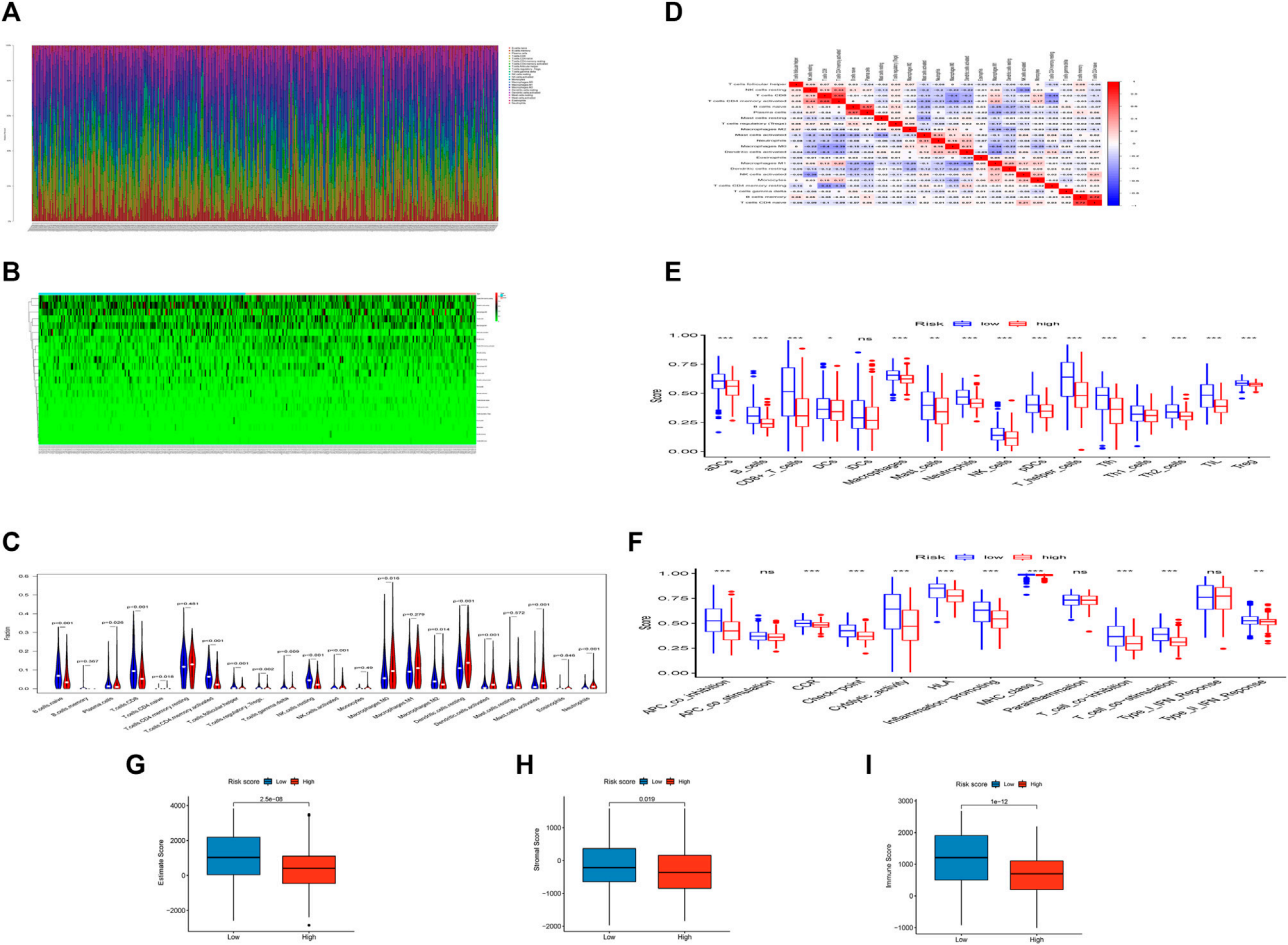
The landscape of the TIME cells in HNSCC and the characteristics of different groups. **(A)**. The proportions of TIME cells of HNSCC patients in CIBERSORT. **(B)**. Heatmap of TIME cells in different subgroups. **(C)**. Violin diagram showing the immune cell composition of different groups in CIBERSORT. **(D)**. Correlation of different immune cells in HNSCC. **(E)**. The proportions of TIME cells of different groups in ssGSEA. **(F)**. The function of TIME cells of different groups in ssGSEA. **(G)**. Boxplot showing differences among different groups in estimate score. **(H)**. Boxplot showing differences among different groups in the stromal score. **(I)**. Boxplot showing differences among different groups in the immune score.

We estimated the tumor immune microenvironment using the CIBERSORT database, ssGSEA, and ESTIMATE. As shown in Figures 5A,B, we mapped the immune landscape for each patient. Sixteen immune cells were remarkably different between the two groups, as shown in the violin plot (Figure 5C). Of the 16 immune cells, 11 kinds of cells were reduced, and five kinds were increased. The infiltration of naïve B cells, plasma cells, CD8 T cells, activated memory CD4 T cells, follicular helper T cells, Tregs, gamma delta T cells, resting NK cells, activated NK cells, and M2 macrophages was lower in the high-risk group. However, CD4 naïve T cells, M0 macrophages, resting dendritic cells, activated dendritic cells, activated mast cells, and neutrophils were more abundant in the high-risk group. We used the CIBERSORT database to explore correlations between immune cells in all HNSCC patients (Figure 5D). T cell memory activation had a high correlation with resting NK cells (correlation coefficient, CC = 0.44) and CD8 T cells (CC = 0.65). Naïve B cells had a high correlation with plasma cells (CC = 0.57). Resting memory T cells had a high correlation with CD8 T cells (CC = −0.44). B cell memory was correlated with CD4-naïve T cells (CC = 0.72). Moreover, we evaluated the correlation between the risk score and immune cells (Table 2) and discovered that naive B cells, CD8 T cells, CD4 memory activated T cells, and resting NK cells had a negative correlation, and activated NK cells, resting dendritic cells, activated dendritic cells, activated mast cells, and neutrophils had a positive correlation.

**TABLE 2:** Correlation of immune cells and risk scores.

Immune cells	Correlation coefficient	*p*-value	Significant
B.cells.naive	−0.1291	0.01345	*
B.cells.memory	−0.00489	0.9256	
Plasma.cells	−0.07281	0.1645	
T.cells.CD8	−0.21213	4.30E-05	***
T.cells.CD4.naive	0.0726	0.1657	
T.cells.CD4.memory.resting	0.000695	0.9894	
T.cells.CD4.memory.activated	−0.19011	0.000254	***
T.cells.follicular.helper	−0.0665	0.2043	
T.cells.regulatory.Tregs	−0.04062	0.4385	
T.cells.gamma.delta	−0.08944	0.0875	
NK.cells.resting	−0.14358	0.005928	**
NK.cells.activated	0.141576	0.006669	**
Monocytes	0.053231	0.3098	
Macrophages.M0	0.075055	0.1519	
Macrophages.M1	0.063393	0.2263	
Macrophages.M2	−0.06687	0.2018	
Dendritic.cells.resting	0.143566	0.005933	**
Dendritic.cells.activated	0.180868	0.000507	***
Mast.cells.resting	0.014909	0.7762	
Mast.cells.activated	0.15005	0.004013	**
Eosinophils	0.03238	0.5369	
Neutrophils	0.131606	0.01173	*

In the ssGSEA, we found that 15 of 16 tumor-infiltrating immune cells (except iDCs) (Figure 5E) and 10 of 13 immune functions (except APC costimulation, parainflammation and type I IFN response) (Figure 5F) were clearly reduced in the high-risk group compared with the low-risk group.

In the integration of CIBERSORT and ssGSEA, most immune infiltrating cells were reduced, such as B cells, neutrophils, and CD8^+^ T cells. This result implied that as the tumor progressed, fewer responsive immune cells infiltrated the tumor, which might lead to the insensitivity of advanced tumors to immunotherapy.

The risk index was conspicuously negatively correlated with the immune, stromal, and ESTIMATE scores, indicating that the infiltration levels of immune and stromal cells decreased with the elevation of the risk score of HNSCC. Higher immune or stromal scores in tumors indicated higher abundances of immune cells or stromal cells. We discovered that patients in the high-risk group had lower stromal, immune, and ESTIMATE scores. Moreover, the higher the score, the better the immune or stromal condition.

### Assessment of Immunotherapy and Drug Sensitivity

**FIGURE 6:**
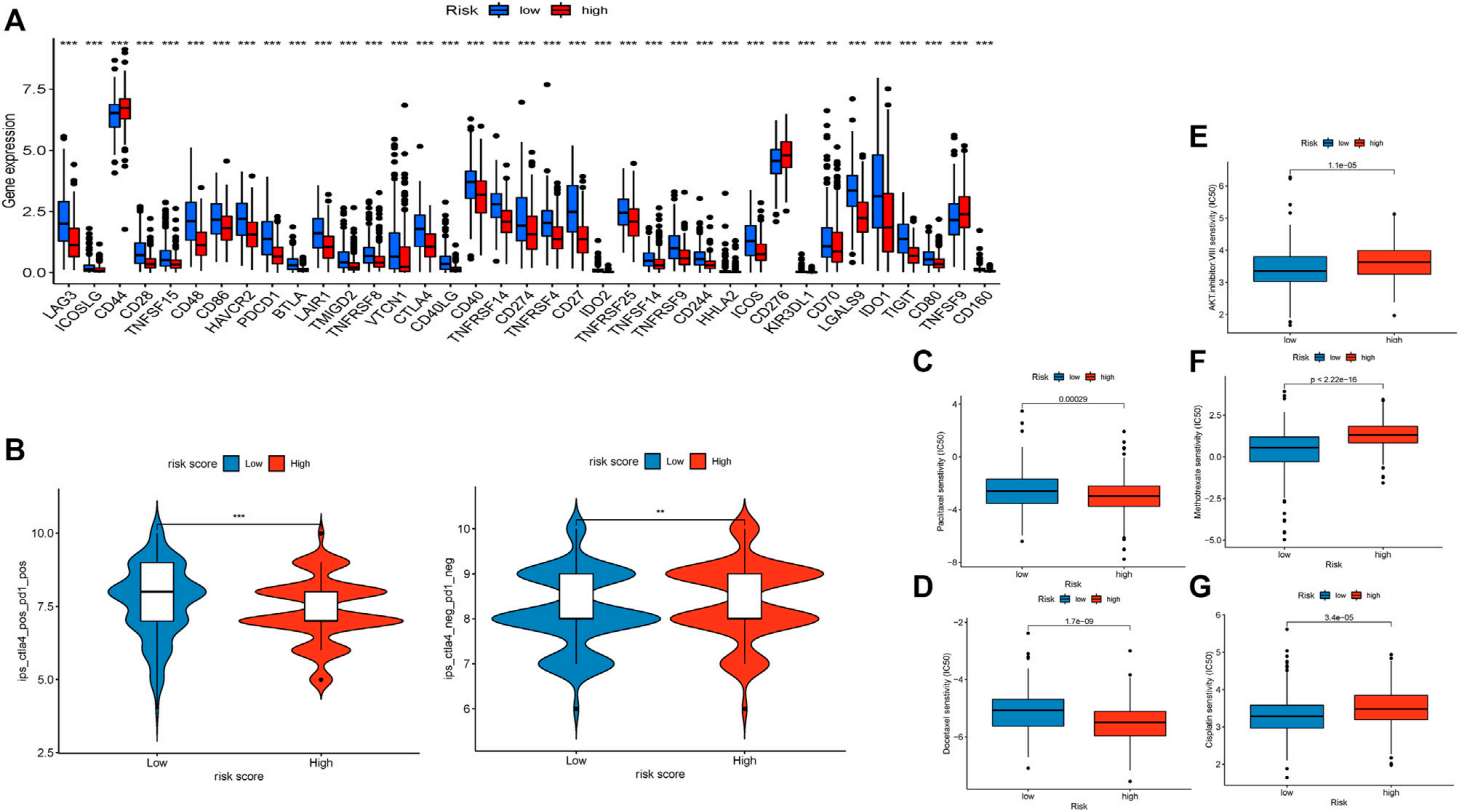
Evaluation of immunotherapy and sensitivity of commonly used chemotherapeutic drugs. **(A)**. Boxplot showing the gene expression of 37 immune checkpoint genes. **(B)**. Violin diagram visualizing the response to anti-CTLA4 and anti-PD-1 therapies between the two groups. **(C)**. Boxplot demonstrating the IC50 of paclitaxel. **(D)**. Boxplot demonstrating the IC50 of docetaxel. **(E)**. Boxplot demonstrating the IC50 of the AKT inhibitor VIII. **(F)**. Boxplot demonstrating the IC50 of docetaxel. **(G)**. Boxplot demonstrating the IC50 of cisplatin.

We discovered that 37 kinds of immune checkpoint genes were notably different (Figure 6A). CD44, CD276, and TNFSF9 were more highly expressed in the high-risk group than in the low-risk group. The expression of 34 other types of genes was the opposite. This result indicated that treatment targets in the high-risk group should differ from those in the low-risk group. We compared anti-PD-1 and anti-CTLA4 therapeutic differences in the two groups via TIDE data (Figure 6B) and found that patients in the low-risk group had better responses.

We compared the differences in the estimated IC50 levels of five chemotherapy drugs, including paclitaxel (Figure 6C), docetaxel (Figure 6D), AKT inhibitor VIII (Figure 6E), docetaxel (Figure 6F), and cisplatin (Figure 6G). Our data revealed higher estimated IC50 levels of paclitaxel (*p* = 0.00029) and docetaxel (*p* = 1.7e-9) in the low-risk group than in the high-risk group. In contrast, the estimated IC50 levels of the AKT inhibitor VIII (*p* = 1.1e-5), docetaxel (*p* < 2.22e-16), and cisplatin (*p* = 3.4e-5) in the low-risk score group were significantly lower than those in the high-risk score group. This result implied that patients with high-risk scores were more sensitive to paclitaxel and docetaxel; patients with low-risk scores were more sensitive to the AKT inhibitor VIII, docetaxel, and cisplatin.

### GSEA

**FIGURE 7:**
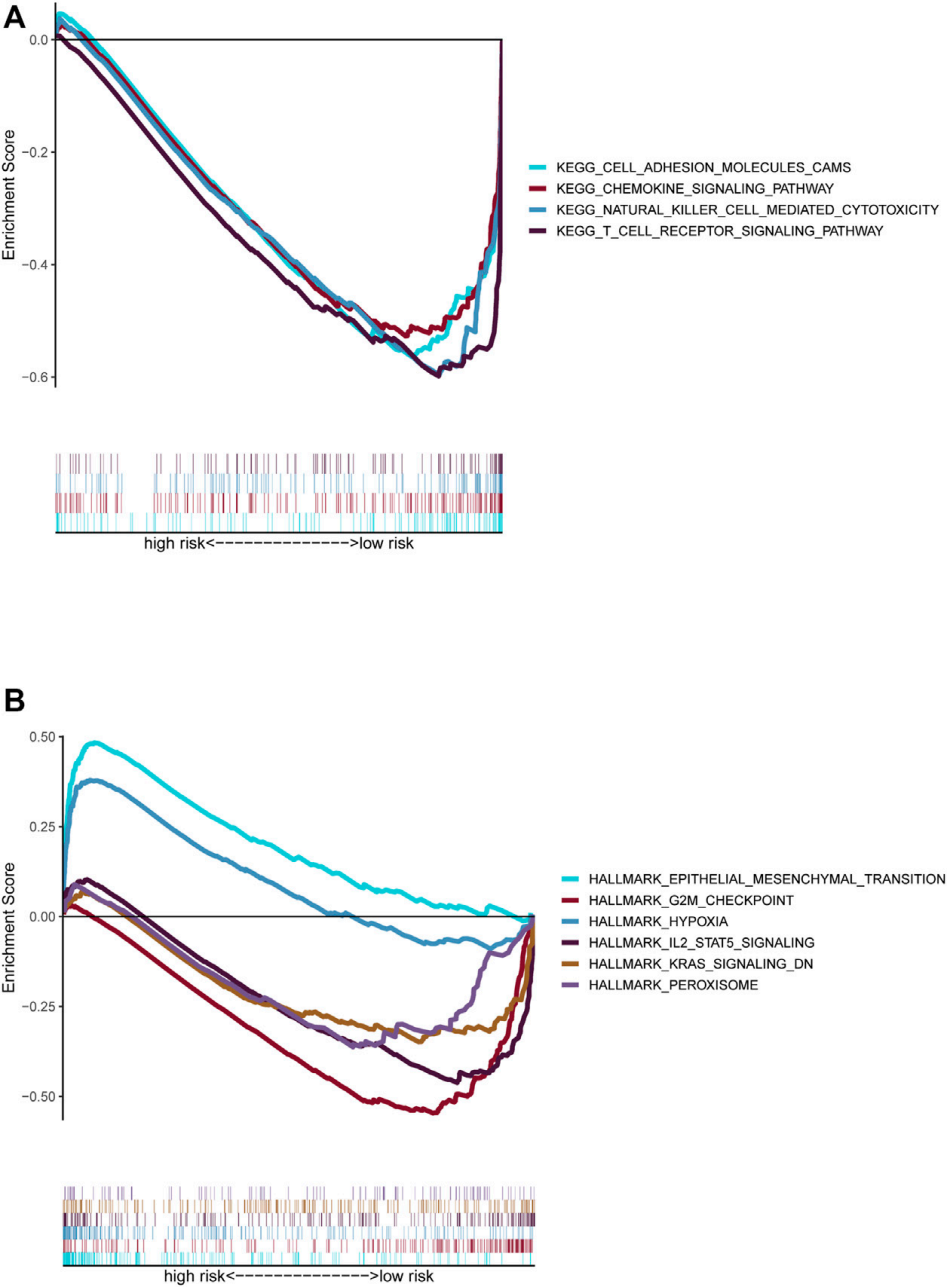
Functional prediction of risk signature via GSEA. **(A)**. The KEGG pathway set was enriched between the high-risk and low-risk groups. **(B)**. The hallmark gene set was enriched between the high-risk and low-risk groups.

GSEA was conducted to compare the high- and low-risk groups. Most of the KEGG enrichment was concentrated in “cell adhesion” and immune-related pathways, such as “chemokine signaling pathway,” “natural killer cell mediated cytotoxicity,” and “T cell receptor signaling pathway,” in the low-risk group (Figure 7A). Moreover, hallmark enrichment was associated with “epithelial-mesenchymal transformation” and “hypoxia” in the high-risk group and “G2M checkpoint,” “IL2 STAT5 signaling,” “KRAS signaling dn,” and “peroxisome” in the low-risk group (Figure 7B).

### The Correlation Between the Prognostic Signature and Somatic Variants

**FIGURE 8:**
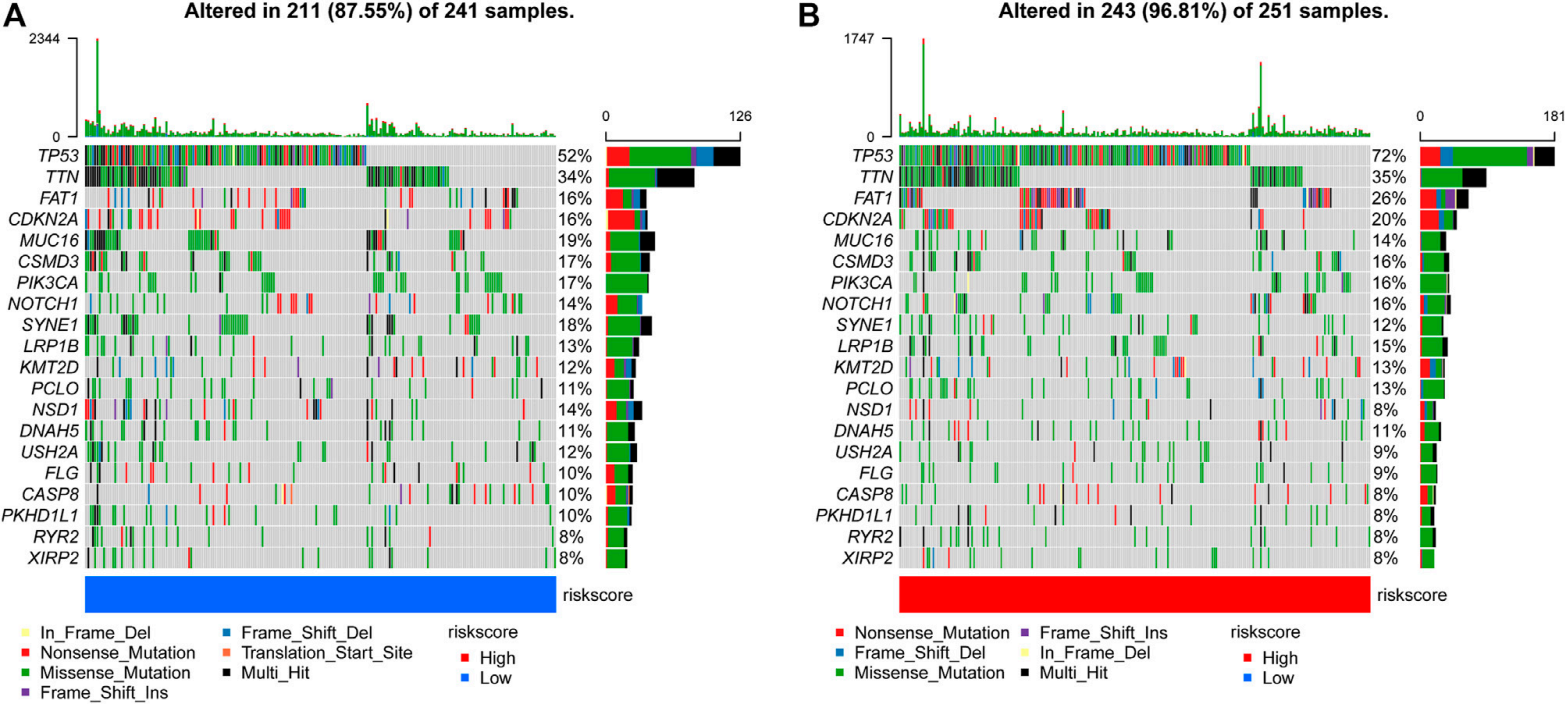
Comparison of mutations in the low-risk group and high-risk group **(A, B)** The top 20 most frequently mutated genes in the low-risk group and high-risk group.

We evaluated the distribution of somatic variants in patients with HNSCC driver genes between the low- and high-risk groups and applied the R package “maftools” to visualize the landscape of mutation profiles in patients with HNSCC(Figure 8). Among all types of mutations, missense mutations accounted for the highest proportion. Our research shows that the alteration frequency of TP53, CDKN2A, and NSD1 was significantly different between the low-risk group and the high-risk group (Table 3).

**TABLE 3:** Differences in mutated genes between high and low risk groups.

Gene symbol	High-risk group		Low-risk group		*p* value	
	Count	%	Count	%		
TP53	175	72	110	52	1.23541E-05	***
TTN	85	35	72	34	0.848289	
FAT1	63	26	34	16	0.010959	**
CDKN2A	49	20	34	16	0.265379	
MUC16	34	14	40	19	0.153097	
CSMD3	39	16	36	17	0.876919	
PIK3CA	39	16	36	17	0.876919	
NOTCH1	39	16	30	14	0.587737	
SYNE1	29	12	38	18	0.06871	
LRP1B	36	15	27	13	0.534907	
KMT2D	32	13	25	12	0.67194	
PCLO	32	13	23	11	0.460061	
NSD1	19	8	30	14	0.028407	*
DNAH5	27	11	23	11	0.942996	
USH2A	22	9	25	12	0.329584	
FLG	22	9	21	10	0.74419	
CASP8	19	8	21	10	0.423731	
PKHD1L1	19	8	21	10	0.423731	
RYR2	19	8	17	8	0.925439	
XIRP2	19	8	17	8	0.925439	

### Prediction Stability and Prediction of Survival Rate

**FIGURE 9:**
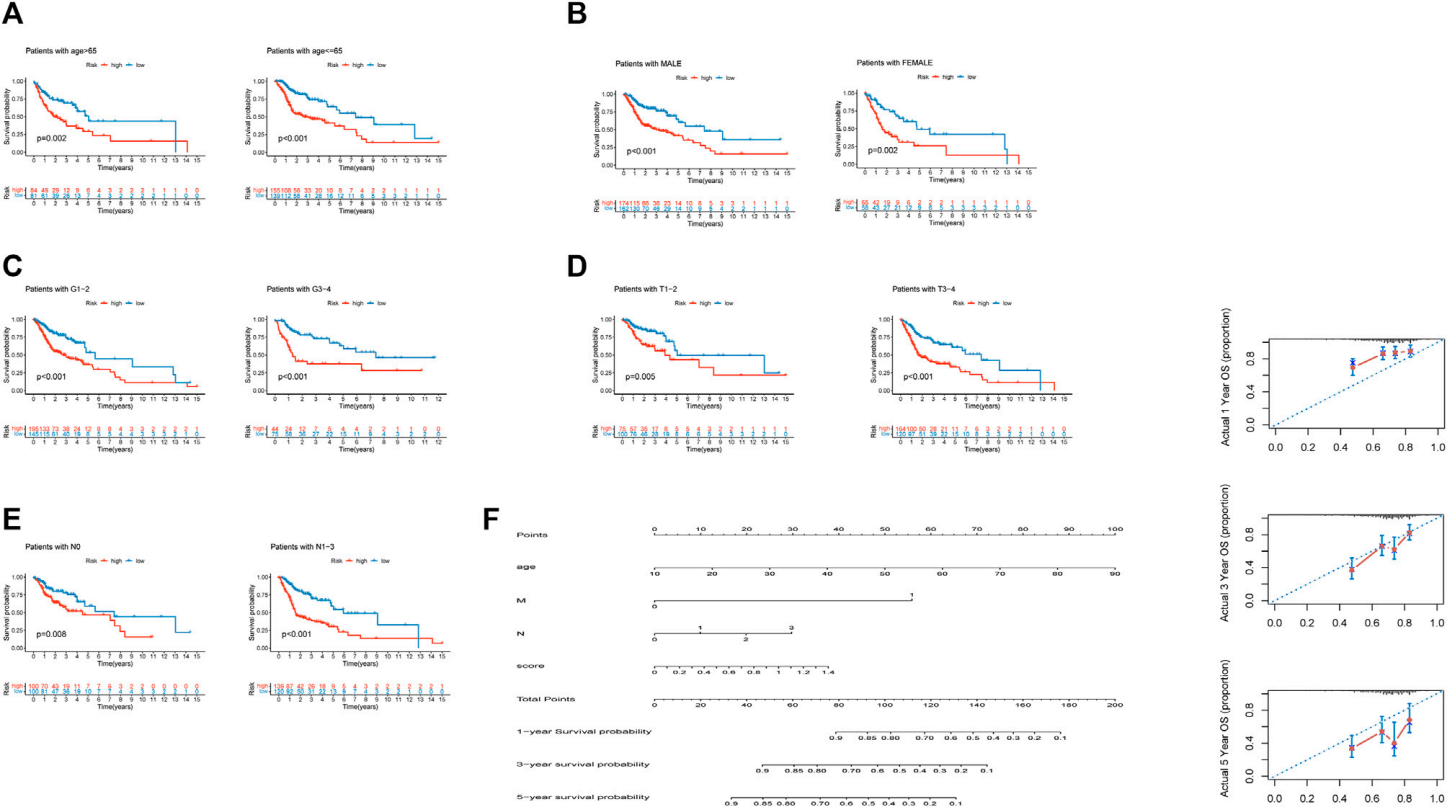
Independence of the model in multiple clinical features and the nomogram prediction of patient survival rate. **(A)**. Independence between differential ages. **(B)**. Independence between differential sexes. **(C)**. Independence between differential grade stages. **(D)**. Independence between differential T stages. **(E)**. Independence between differential N stages. **(F)**. Nomogram predictions of 1-, 3-, and 5 years survival in HNSCC patients.

We found that our risk signature had high stability among different clinical subgroups, such as age, sex, grade, T stage, and N stage, *via* Kaplan–Meier survival curves (Figures 9A–E). Demographic data from TCGA patients were presented in Table 4. We established a nomogram including age, M stage, N stage, and risk score to predict 1, 3, and 5 years survival rates (Figure 6F). We found that the nomogram corresponded fairly with the actual survival rate.

**TABLE 4:** Demographic data from TCGA patients.

			High risk		Low risk	
			N	%	N	%
Age (Years)			254		245	
	≥65	249	112	44.1	137	55.9
	<65	250	142	55.9	108	44.1
Gender						
	Male	366	184	72.4	182	74.3
	Female	133	70	27.6	63	25.7
Grande						
	1	61	37	14.6	24	9.8
	2	298	167	65.7	131	53.5
	3	119	44	17.3	75	30.6
	4	2	0	0.0	2	0.8
	X	16	5	2.0	11	4.5
	Unknown	3	1	0.3	2	0.8
Stage						
	1	25	9	3.5	16	6.5
	2	79	37	14.6	42	17.1
	3	89	43	16.9	46	18.8
	4	303	165	65	138	56.3
	Unknown	3	0	0.0	3	1.2
T						
	0	1	0	0.0	1	0.4
	1	47	15	5.9	32	13.1
	2	148	64	25.2	84	34.3
	3	114	60	23.6	54	22.0
	4	184	111	43.7	73	29.8
	X	4	3	1.2	1	0.4
	Unknown	1	1	0.4	0	0.0
M						
	0	484	247	97.2	237	96.7
	1	4	3	1.2	1	0.4
	X	11	4	1.6	7	2.9
N						
	0	212	106	41.7	106	43.3
	1	75	35	13.8	40	16.3
	2	197	105	41.3	92	37.6
	3	9	5	2.0	4	1.6
	X	5	2	0.8	3	1.2
	Unknown	1	1	0.4	0	0.0
Race						
	AI/AN	2	1	0.4	1	0.4
	Asian	10	5	2.0	5	2.0
	Black	47	27	10.6	20	8.2
	White	426	212	83.5	214	87.3
	Unknown	14	9	3.5	5	2.0
HPV						
	Negative	72	33	13.0	39	15.9
	Positive	30	3	1.2	27	11.0
	Unknown	397	218	85.8	179	73.1
Smoke						
	Yes	378	191	75.2	187	76.3
	No	111	56	22.0	55	22.4
Sample Type		10	7	2.8	3	1.2
	Primary	499	254	100.0	245	100.0
	Metastatic	0	0	0.0	0	0.0

AI/AN, American Indian or Alaska native.

### Verification of Expression Differences

**FIGURE 10:**
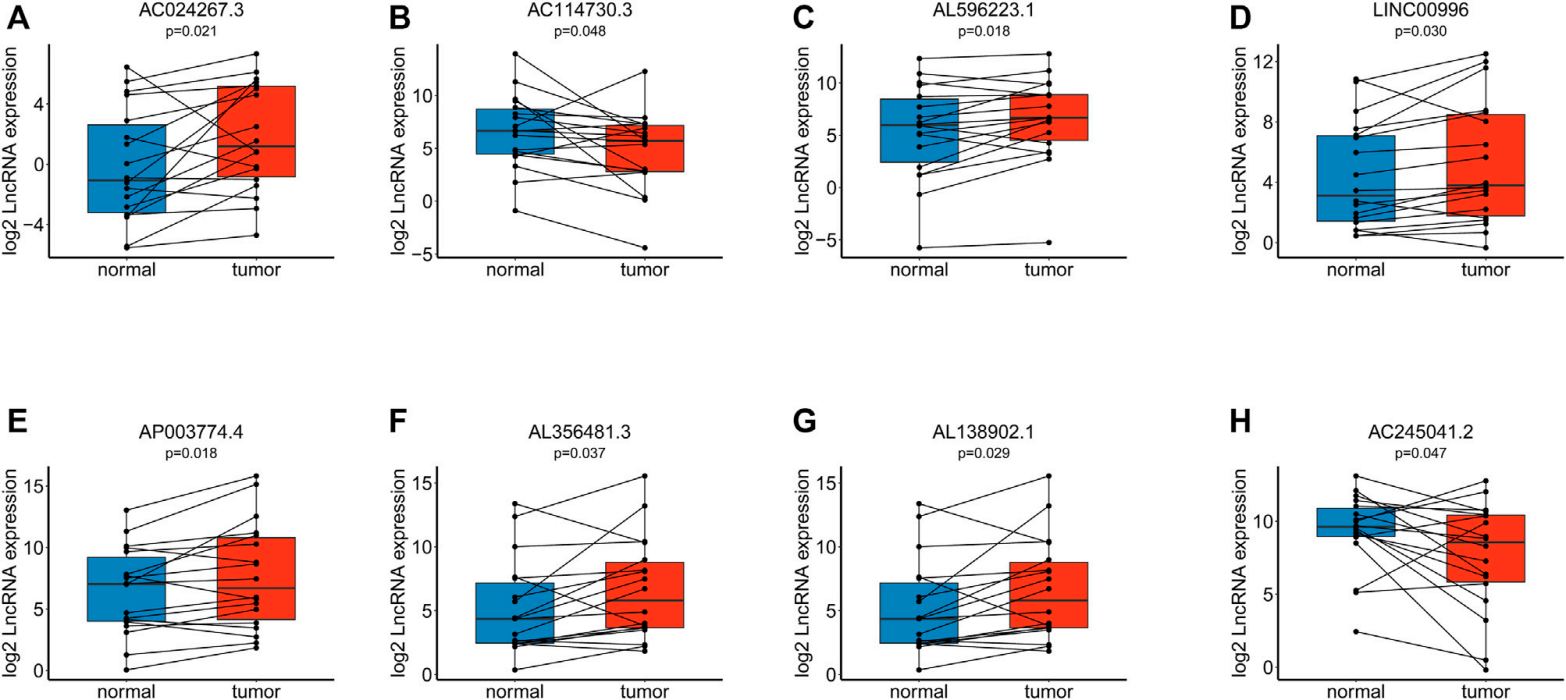
Verification of the model via RT–qPCR among eighteen paired tissues. **(A)**. Expression of AC024267.3. **(B)**. Expression of AC114730.3. **(C)**. Expression of AL596223.1. **(D)**. Expression of LINC00996. **(E)**. Expression of AP003774.4. **(F)**. Expression of AL356481.3. **(G)**. Expression of AL138902.1. **(H)**. Expression of AC245041.2.

Using RT–qPCR, we found that eight lncRNAs included in our signature had distinctive expression differences between eighteen paired paracarcinoma tissues and cancer tissues. AC024267.3 (*p* = 0.021) (Figure 10A), AL596223.1 (*p* = 0.018) (Figure 10C), LINC00996 (*p* = 0.030) (Figure 10D), AL356481.3 (*p* = 0.037) (Figure 10F), and AL138902.1 (*p* = 0.029) (Figure 10G) were upregulated in tumors compared with normal tissues. In addition, AC114730.3 (*p* = 0.048) (Figure 10B), AP003774.4 (*p* = 0.018) (Figure 10E), and AC245041.2 (*p* = 0.047) (Figure 10H) were downregulated.

## Discussion

EMT is a process in which epithelial cells acquire mesenchymal cell characteristics and is closely related to tumor invasion, metastasis, and chemotherapy resistance in tumor progression (Lamouille et al., 2014; Pastushenko and Blanpain, 2019; Xing et al., 2019; Guo et al., 2020). The molecular mechanisms involved in the transition from an epithelial phenotype to a mesenchymal state are complex and controlled by multiple signaling pathways (Gugnoni and Ciarrocchi, 2019). Recently, more and more evidence has emphasized the regulatory role of lncRNA in the tumor EMT process (Grelet et al., 2017; Chen and Shen, 2020; Hu et al., 2020; Peng et al., 2020).

In this paper, EMT-related lncRNAs were comprehensively identified in HNSCC for the first time, and an EMT-related lncRNAs prognostic signature was established and validated. Then, we evaluated the relationships of the prognostic signature with tumor immune microenvironment characteristics, somatic variants, and chemotherapy. Finally, we verified lncRNA expression differences among eighteen paired paracarcinoma tissues and cancer tissues.

The Pearson's correlational coefficients were used to identify EMT-related lncRNAs. Univariate Cox regression was used to search for EMT-related lncRNAs with prognostic values. LASSO regression analysis was used to construct a predictive model to identify patients as a high-risk and low-risk group (Figure 2). The AUC values of training set and test set confirm that our signature had medium accuracy for evaluating the prognosis of HNSCC patients (Figures 3E–O). Univariate and multivariate Cox regression analyses demonstrated that the risk score was an independent predictor of prognosis in HNSCC patients (Figures 4A,B). Additionally, PCA showed that the high-risk and low-risk groups were distributed in different directions, suggesting significant differences in immune status between the high-risk and low-risk groups (Figures 3P–S).

ESTIMATE, a tool based on gene expression signatures, was used to predict the proportions of immune cells and stromal cells in tumors (Yoshihara et al., 2013). The high-risk score group had higher tumor purity and lower stromal and immune scores than the low-risk group. Moreover, we found that patients with high stromal and immune scores had shorter survival times, indicating that high-risk scores could predict a poor prognosis for HNSCC patients. This finding ultimately indicated that EMT could be related to the reconstruction of the tumor microenvironment while affecting tumor growth and progression (Li et al., 2020; Xu et al., 2021a; Guo et al., 2021; Lin et al., 2021). EMT is a critical event in the process of tumor metastasis, and our results support this conclusion. There were also differences between the two groups in terms of the tumor immune microenvironment. The presence of inhibitory immune cells was higher in the high-risk group, and the proportion of effector cells was higher in the low-risk group, suggesting that the outcome of immunotherapy might be better in the low-risk group. Many factors impact the effect of immunotherapy in HNSCC. Despite the favorable clinical benefits of immunotherapy, only a portion of HNSCC patients show a response to immunotherapy. Using our signature, we found that the sensitivity of PD-1 and CTLA4 was clearly different in the high- and low-risk groups. Patients in the low-risk group responded to anti-PD-1 and anti-CTLA4 drugs. It is worth noting that TIDE is built based on data from melanoma and NSCLC cancers. Although it has been proved that TIDE score can be used to predict HNSCC in a lot of literature, its accuracy is still worth considering. We found that patients with high risk scores were more sensitive to paclitaxel and docetaxel. Meanwhile, patients with low risk scores were more sensitive to the AKT inhibitor VIII, docetaxel, and cisplatin. Based on these findings, clinicians can implement comprehensive and individualized treatment plans for patients.

The GSEA results showed a significant connection between the EMT-related LncRNA signature and the tumor immune microenvironment, including chemokines, natural cells, and T cells. Hallmarks of EMT and the “hypoxia” pathway enriched in the high-risk group promote tumor development and metastasis, and “G2M checkpoint”-related gene overexpression and deficiency of KRAS inhibition-related genes promote abnormal cell division in different tumors (Mittal, 2018; Shirazi et al., 2020; Smith et al., 2020). The peroxisome generally exhibits dysfunction in tumors, especially advanced tumors (Islinger et al., 2018).

Growing evidence indicates that TP53 mutations are potential predictors to immunotherapy (Caponio et al., 2020; Zhang et al., 2020). Meanwhile, the overall survival of TP53 mutant-type (TP53 MT) patients is worse than that of TP53 wild-type (TP53 WT) patients (Wu et al., 2020a). Our research shows that the alteration frequency of TP53, CDKN2A, and NSD1 was significantly different between the low-risk group and the high-risk group (Figure 8; Table 3).

Some of these genes have been reported in a variety of cancers. Our results are consistent with the conclusions of the existing articles. AC114730.3 is a protective factor for patients with HNSCC and bladder cancer. The higher the AC114730.3 expression, the better the prognosis (Wu et al., 2020b; Xu et al., 2021b). AC245041.2 could influence transcription factors via LAMA3 and then alter the expression of other protein molecules. Furthermore, the high expression of AC245041.2 supports the survival of patients with clear cell renal cell carcinoma (Tian et al., 2021). Wang et al. also similarly reported that AC245041.2 was a protective factor in clear cell renal cell carcinoma (Wang et al., 2019b). Lina et al. reported that LINC00996 was mainly involved in biological processes such as vascular development, positive metabolic processes, and growth regulation (Lina, 2021). Ge H et al. reported that low expression of LINC00996 was related to metastasis and a low survival rate. In this process, the JAK-STAT, NF-κB, HIF-1, TLR and PI3K-AKT signaling pathways might have a crucial effect (Ge et al., 2018).

Our study lays the foundation for further research into EMT-related lncRNAs and has profound implications for screening criteria for patients receiving immunotherapy. The original EMT-based classification could provide prognostic predictors for HNSCC and might guide clinicians in selecting potential patients who respond to immunotherapy for the preferential use of immunotherapy. This study still has a few limitations. First, our model may have some deviation due to the use of TCGA data and not external data. Although we only used TCGA data, univariate and multivariate Cox analyses, grouping modeling, stratified prognosis analyses, and ROC curves all indicated that our model was stable and accurate. Second, further prospective external verification with a large sample of HNSCC patients from our hospital is required. Future functional studies are essential to clarify the underlying mechanism of EMT-related lncRNAs in HNSCC.

## Conclusion

In summary, we constructed and verified the independent predictive value of an EMT-related lncRNA-based prognostic signature for patients with HNSCC for the first time. More importantly, these results provide important references for personalized chemotherapy and immunotherapy.

## Data Availability

The datasets presented in this study can be found in online repositories. The names of the repository/repositories and accession number(s) can be found in the article/Supplementary Material.
