# Supplementary material for: Identification and Validation of an EMT-Related LncRNA Signature for HNSCC to Predict Survival and Immune Landscapes
Source: Front Cell Dev Biol. 2022 Jan 20;9:798898. doi: 10.3389/fcell.2021.798898 (PMC8902443; doi:10.3389/fcell.2021.798898)
Supplement: Supplementary file 1 [file Table1.DOCX]

Stable.1 Information of patients used to verify differential expression

| Name | Age (Years) | Gender | Grade | Stage | T | M | N | Smoke | Sample Type | Diseased Region |
| --- | --- | --- | --- | --- | --- | --- | --- | --- | --- | --- |
| ZW | 55 | Male | 3 | 4 | 4 | 0 | 2 | Yes | Primary | Left Cheek |
| MH | 76 | Female | 3 | 4 | 3 | 0 | 2 | No | Primary | Left Gingiva |
| QT | 71 | Male | 3 | 4 | 4 | 0 | 1 | Yes | Primary | Left Cheek |
| XY | 69 | Male | 2 | 4 | 1 | 0 | 2 | No | Primary | Left Cheek |
| DW | 79 | Male | 3 | 4 | 2 | 0 | 2 | Yes | Primary | Right Tongue |
| ZL | 52 | Male | 3 | 4 | 3 | 0 | 2 | Yes | Primary | Oropharynx |
| XG | 69 | Male | 3 | 4 | 4 | 0 | 2 | No | Primary | Right Cheek |
| SZ | 60 | Female | 3 | 2 | 2 | 0 | 0 | No | Primary | Right Gingiva |
| TZ | 65 | Male | 3 | 4 | 3 | 0 | 2 | Yes | Primary | Left Cheek |
| LM | 72 | Female | 3 | 4 | 3 | 0 | 3 | Yes | Primary | Left Gingiva |
| HL | 48 | Male | 2 | 4 | 3 | 0 | 2 | No | Primary | Right Cheek |
| PL | 52 | Male | 2 | 3 | 3 | 0 | 1 | Yes | Primary | Right Tongue |
| JL | 63 | Female | 3 | 3 | 3 | 0 | 0 | No | Primary | Upper lip |
| MW | 57 | Male | 2 | 4 | 3 | 0 | 2 | Yes | Primary | Left Tongue |
| BP | 55 | Female | 2 | 4 | 3 | 0 | 1 | No | Primary | Left Gingival |
| SL | 70 | Male | 2 | 1 | 1 | 0 | 0 | Yes | Primary | Right Tongue |
| QW | 64 | Male | 2 | 3 | 2 | 0 | 1 | Yes | Primary | Right Cheek |
| XC | 71 | Male | 2 | 4 | 4 | 0 | 2 | No | Primary | Right Tongue |
